# This I believe in genetics: discovery can be a nuisance, replication is science, implementation matters

**DOI:** 10.3389/fgene.2013.00033

**Published:** 2013-03-14

**Authors:** John P. A. Ioannidis

**Affiliations:** ^1^Department of Medicine, Stanford Prevention Research Center, Stanford University School of MedicineStanford, CA, USA; ^2^Department of Health Research and Policy, Stanford Prevention Research Center, Stanford University School of MedicineStanford, CA, USA; ^3^Department of Statistics, Stanford University School of Humanities and SciencesStanford, CA, USA

For several years now, accumulation of genetic information has accelerated at a pace that exceeds the acceleration of computer capacity (Moore's law) and there is no discernible limit to prospects of further growth. As the cost per unit of obtained genetic information is plummeting (Niedringhaus et al., 2011), genetics has become a frontrunner and a catalyst of the informatics revolution that is affecting very diverse biomedical scientific fields. In non-biomedical science, somewhat equivalent roles to genetics have been assumed by other disciplines that are also driven by big data, e.g., observational astrophysics and high-energy and particle physics. This evolving big data paradigm offers the opportunity to re-think about priorities surrounding different steps in the scientific process.

Making new discoveries has been deemed traditionally the most important aspect of scientific investigation. By “traditionally,” I mean the usual criteria of funding agencies, the publication priorities of major scientific journals, the selection processes for prestigious academic recognitions, even the public imagination and fantasizing on what scientific investigation is all about. According to the most widespread cliché, scientists discover new things by collecting and analyzing more and more data. However, the genomic information explosion has caused an oversupply crisis. This crisis has drastically devalued the currency of discovery. Data are overabundant; most of it can be accumulated without any serious thinking; actually researchers with personal mental labor are not even needed to collect data: commercial chips do the trick, and robots do the pipetting. Not only data are abundant, discoveries are also as abundant. Even if we postulate an 1:1,000,000 ratio of claimed discoveries to data items, there are zillions of discoveries that can now be claimed every day. Based on what we have started to surmise empirically, most of these claimed discoveries are likely to be either totally false preliminary observations (Ioannidis, 2005) or substantially exaggerated results (Ioannidis, 2008), a consequence of the extreme multiplicity of the probed data-space, the winner's curse (Zollner and Pritchard, 2007), and other biases. “Negative” results have almost disappeared from many scientific fields, especially those with “softer” measurements and more flexible analytical tools (Fanelli, 2010). Results procured by the most popular research sub-fields seem to have the lowest reliability (Pfeiffer and Hoffman, 2009). It seems likely that there is an extraordinary large number of small, weak effects and links (“risks” in epidemiological language), barely discernible from measurement error and diverse potential biases. Single discoveries made in single databases are likely to mean very little, they are mostly a nuisance that propagates confusion in the literature. Exceptions certainly occur, and some strong/large effects may still exist, awaiting discovery. Even then, it is unlikely that the discoverer who hits upon them will have any more merit than the thousands of other researchers who only come across the flooding multitude of weak or false effects. The process of rewarding discoverers claiming large effects (be that with grants, tenure, or Nobel prizes) may eventually become indistinguishable from running a lottery. If we add human nature, biases, and conflicts (Ioannidis, 2011), a lottery system may be even preferable.

In settings where claimed discoveries become more than we can absorb and tolerate and when most claims about discoveries are false, replication becomes the most important, central piece of science. Replication efforts typically require a shift toward team science (e.g., consortia; Austin et al., 2012). They place emphasis on a community effort to find the few true among many wrong proposed leads. Replication offers a realistic chance of maintaining the scientific literature reasonably noise-free. Genetics has shown clearly how important this is. Human genome epidemiology was radically transformed in the last decade by the adoption of a rigorous replication culture. While the vast majority of claims for genetic associations based on biological plausibility speculations and performed by single teams without replication were apparently wrong (Ioannidis, 2011), large meta-analyses of genome-wide association studies using agnostic platforms and *sine-qua-non*, rigorous replication across multiple teams and multiple datasets has yielded thousands of associations with unquestionably high credibility (Hindorff et al., 2009). How many other scientific fields are still conducting studies based on biological plausibility speculations and performed by single teams without replication? Probably most of the literature in diverse fields has been based on these same premises and will likely collapse once rigorous replication practices are adopted.

As replication creates an expanding, more reliable basis of knowledge, the need to further translate and implement this knowledge becomes also essential. Until now, research emphasis (and funding) has been placed disproportionately on T0 (discovery research) and some T1 (research for development of new tests or therapies) (Schully et al., 2011), with exponentially diminishing investments as we move to later stages of translation. I have great sympathy for the concept of science for the sake of science. Perhaps much scientific knowledge has absolutely no practical use and no further translational potential. This would not diminish its importance. For genetics in particular, the satisfaction of intellectual curiosity for the labyrinth of genetic architecture is a legitimate goal. However, many opinion leaders argue (to colleagues, politicians, and taxpayers) that emphasis on genetics (or other) research is justified because of the practical potential that this knowledge may have for improving health outcomes in single individuals and larger populations (Feero et al., 2010). This promise is in stark disagreement with the scant resources that are currently applied to later stages of translational research and, in particular, on the potential implementation of the accumulating research findings. Accomplishing these translational stages will require rigorous methods, including well-performed clinical trials. The expectation that real progress will happen and genetics will change our everyday life for the better in a vacuum of rigorous implementation evidence is not realistic. Genetics can revolutionize medicine and drastically improve outcomes, or may lead to the adoption of millions of genetics-based tests and interventions that are false, useless, costly, or all of that. We have had so many brilliant, spectacular, innovative discoveries so far—more of the same brilliant, spectacular innovation alone is becoming terribly boring; it is rigorous replication that guarantees science and it is successful translation and implementation that matters.
